# Defining Leadership in Smart Working Contexts: A Concept Synthesis

**DOI:** 10.3389/fpsyg.2020.556933

**Published:** 2020-09-16

**Authors:** Michela Iannotta, Chiara Meret, Giorgia Marchetti

**Affiliations:** ^1^Department of Management, Sapienza University, Rome, Italy; ^2^Faculty of Economics, Sapienza University, Rome, Italy

**Keywords:** leadership, smart leadership, smart working, e-leadership, concept synthesis

## Abstract

This paper begins by considering the importance of leadership to pursue behavioral, cultural, technological, and ethical aspirations of smart working practices in order to develop a comprehensive understanding of leadership in smart working contexts. We adopt a literary approach to the concept synthesis method with which to critically analyze and conceptually map a wide set of related notions of leadership that are connected with the evolutive dynamics of smart working approaches. According to the scope conditions of the research, the role of leadership emerges with the purpose of changing behaviors, creating shared meanings, and integrating physical and technology-mediated interactions in smart working environments. With this in mind, the iterative integration of smart working and leadership literature has gradually begun to detect and classify the main characteristics of leadership in smart working contexts in terms of leadership antecedents, attributes, and outcomes. The interpretative synthesis results in an overarching conceptualization of “leading in smart working contexts” that depicts leadership as a naturally emerging phenomenon that combines agile logics and change management practices to align interests at different levels of the organization. These premises lead to the alleged “triple-win” configuration of smart working approaches. While encouraging in-depth discussion about the facilitative and performative function of leadership in smart working contexts, this study contributes to advancing knowledge on what “being a smart leader” actually means, and how to operatively apply this notion in smart working contexts. Together, the concept delineation and the operational definition of “smart leadership” offer important insights for both managerial action and future directions of research.

## Introduction

Recent socioeconomic and technological changes in business environments have enabled new ways of working based on flexible work arrangements and an extensive use of information technologies that support employees to potentially work in any time and space (De Leede and Heuver, 2016). Such approaches are generally referred as “smart working” practices (Boorsma and Mitchell, 2011; Gastaldi et al., 2014; Zheltoukhova, 2014; McEwan, 2016). More exhaustively, smart working has been defined as an agile and dynamic way of working that leads to high performance, increased productivity, and improved job satisfaction that result is a “triple-win” configuration for customers, employees, and organizations (Gastaldi et al., 2014; Zheltoukhova, 2014; McEwan, 2016). To that regard, many scholars have indicated a new paradigm shift that “is being driven by extreme changes in approaches to work, work cultures, business architectures, premises, decision making, communications, and collaboration” (Boorsma and Mitchell, 2011, p. 2). Others, more cautiously, have made reference to evolutionary changes in work and management practices that are enabled by technological advances and that foster both organizational agility and new workforce expectations (e.g., Zheltoukhova, 2014; McEwan, 2016; Bednar and Welch, 2019). Indeed, the application of smart working requires interventions across the organizational structure, workplace layout, work practices, and human behavior levels. From a change management perspective, this entails the aim of building a collaborative ecosystem in which trust, flexibility, autonomy, and employee skills have primacy (Zheltoukhova, 2014; McEwan, 2016). Such an environment requires a profound cultural evolution, which can only take place when it is endorsed by a broader intervention in management and leadership capabilities. As noted by McEwan (2016), such capabilities are resources that help leaders to instigate change in smart working contexts. In this vein, the Chartered Institute of Personnel and Development has suggested that when organizational and technological changes are not aligned with leadership interventions, individuals fail to adopt new behaviors as they are still anchored to the old organizational rules (Zheltoukhova, 2014). This circumstance could strongly undermine the triple-win configuration of smart working.

While it is clear that cultural changes require guidance, and that the notion of smart working brings to mind that of smart leadership, extant literature has not addressed this latter concept with explicit reference to smart working contexts. A first definition of smart leadership was provided by Finkelstein and Jackson (2005) as one of the main pillars of a smart organization. According to Finkelstein and Jackson (2005), smart leaders are authentic leaders, as they clearly exhibit their own set of values and act with deep responsibility toward shareholders to guarantee effective alignment between personal and organizational interests. Similarly, Singh (2017, p. 1) underlined the importance of smart leadership in creating a participated view of change, asserting:

smart leadership is about being agile and flexible. It lies in creating an exciting and compelling vision and inspiring people to deliver on it. It energizes people and ignites to unleash and exploit their talents. It focuses on imbuing a team with wisdom from experience and knowledge. A calm and rational demeanor even in the face of storms, volatility and uncertainty, characterizes smart leadership. It is forward thinking and anticipatory.

Finally, Rao (2013) suggested that smart leadership is about finding a balance between soft power (i.e., achieving outcomes by attracting and persuading), and hard power (i.e., using rewards or punishments).

From the literature we reviewed, the notion of “smart leadership” appears to be composite and multidimensional. It has often been intended as a set of characteristics that essentially belong to other extant constructs, most of which are related to the transformational view of leadership (Bolognini, 2019). Moreover, the existence of many other smart working–related concepts, such as agile leadership, e-leadership, and leadership 4.0, does not facilitate identification of the boundaries of what “leading in smart working contexts” actually signifies. In turn, with respect to professionals asking academics what they understand leadership in smart working contexts to mean, we were unable to find a unique definition that could be expressly adopted in practice. On the one hand, this gap may be due to a broader lack of theorization as identified by Cortellazzo et al. (2019) in their review of literature about the impact of technology on leadership. On the other hand, the proliferation of empirical contributions has provided a range of notions on which we can draw, albeit without well-established and consensual definitions that would contribute to a clear delineation of the boundaries among them (Cortellazzo et al., 2019).

Overall, what emerges from these arguments is that the extant literature still lacks contributions that delineate a unique notion of leadership explicitly applied to smart working contexts. To fill this gap, the current paper proposes a synthetic integration of both smart working and leadership literature, aimed at answering the following research questions: (1) What kind of leadership should be enacted to facilitate the development of a smart working philosophy? (2) What kinds of attributes, skills, and capabilities are needed for leading in smart working contexts? and (3) What distinguishes smart leadership from related concepts? After processing competing notions of leadership, and by connecting them to the evolutive dynamics of change required by smart working, our concept synthesis results in a comprehensive understanding of how leadership capabilities can create environments conducive to enhancing smart working practices.

The remainder of this paper is structured as follows: the next section describes the research rationale and methods adopted in conducting this conceptual analysis. After delimiting the boundaries of the field by referring to a wide range of sources, the progressive process of concept delineation is described, and a categorization of those antecedents, attributes, and outcomes of leadership that fit smart working dynamics is provided. Results of the concept synthesis are then presented and discussed by highlighting academic contributions, managerial implications, and future directions of research.

## Research Rationale and Methods

According to Morse et al. (1996, p. 256), a concept is considered “mature” when it is “relatively stable, clearly defined, with well described characteristics, demarcated boundaries, specified preconditions and outcomes.” However, Morse et al. (1996) suggest that, although a consistent definition exists, some concepts exhibit a number of inconsistencies in their application into the research field. Concept analysis is particularly useful when the aim is to identify attributes that constitute the concept, to better refine a concept, or to evaluate competing concepts in terms of their relations to the phenomenon under investigation (Morse et al., 1996; Rodgers, 2000).

Since the objective of our investigation covers fields comprising variant definitions and continuous updates, we conducted a conceptual analysis in order to find similarities and discrepancies among sets of related concepts (Walker and Avant, 1994). Our rationale is that attributes for leading in smart working contexts cannot be delineated without strict reference to the evolutive dynamics that occur in such contexts. Therefore, in line with Morse et al. (1996), our conceptual analysis is motivated by two main principles. The first is a logical principle, as we aim to coherently and systematically analyze concepts in relation to each other. The second is a pragmatic principle, as we aim to delineate concepts that might be operationalized and effectively used in managerial action (Rodgers, 2000).

Starting from Wilson’s (1969) and Walker and Avant’s (2005) procedures for concept synthesis, we organized our study around four macro steps: (1) delimitation of the field, (2) collection of sources, (3) clustering into smaller units, and (4) evaluation. Concept synthesis was performed according to a literary approach (Walker and Avant, 2005); that is, the analysis of concepts is based on the study of published literature. This approach allowed us to critically review the main sources of interest, to iteratively search for notions and meanings across documents, and to map the key concepts for inclusion in the analysis. This entailed conceptually mapping the literature, including ongoing research, and scientific and practitioner articles, as well as synthesizing findings from various types of studies (Anderson et al., 2008; Grant and Booth, 2009). The iterative design of this methodological approach means that the boundaries between macro-phases are not strictly fixed.

In line with Levac et al. (2010), at the beginning of the process the researchers met to discuss inclusion and exclusion criteria for the documents selected for the study. Inclusion criteria were the relevance, importance, usefulness, and purposiveness of the source in relation to the role of leadership in smart working contexts. Then, each of the three authors independently reviewed abstracts and contents for inclusion.

To delimitate the field of study, we started by analyzing smart working literature by selecting information that was simultaneously interesting, relevant, important, and useful for the research purpose. The aims of this initial analysis were to (1) capture the main dynamics around which the smart leadership concept might be delineated, and (2) explore the potential link between smart working features and applicable notions of leadership. The three authors performed this delimitation analysis separately by summarizing recurring themes in a standardized format. The resulting topics that point out the role of leadership in smart working contexts were: (1) changing behaviors, (2) creating shared meanings in the change management process, and (3) integrating physical and technology-mediated interactions.

Starting from these insights, we proceeded to select from the literature notions of leadership that could fit the abovementioned dimensions for the iterative process of literary analysis. The aim of this second step of the concept analysis was to identify further insights into the different notions of leadership at hand (i.e., participative, transformational, organic, catalyst, purposeful, ethical, and resonant leadership; e-leadership; leadership 4.0; etc.) and to obtain a comprehensive understanding of smart leadership specifically conceived for smart working contexts. We continued to collect sources until sufficient saturation and coverage was reached (Nuopponen, 2010a). The purpose was to determine what qualifies (or does not qualify) as a defining attribute of leadership in smart working contexts, and which characteristics or attributes best fit the concepts of interest. Thus, the first two steps concluded with a definition of the field and its delimitation within a domain, or identification of the number of concepts to be addressed during the conceptual synthesis.

The third step consisted of a full analysis of the collected sources to identify and cluster categories and subcategories related to the concept under investigation. In this way, the main characteristics of leadership in smart working contexts were detected and classified in terms of such leadership’s antecedents, attributes, and outcomes. More exhaustively, attributes represent characteristics that are mostly frequently associated with the concept, while antecedents are events, criteria, or conditions that allow the concept to occur. Outcomes are generally referred to as the consequences that are produced by both antecedents and attributes of the concept (Walker and Avant, 2005). To support the process of concept delineation, the authors reciprocally asked each other questions about the categories at hand, thus converging in a unique shared framework. Operationally, terminological analysis of concepts considers the fact that these concepts may entail synonyms, polysemes, and homonyms (Walker and Avant, 1994; Nuopponen, 2010b). In practice, this represents the bridge leading to the next stage. In line with Nuopponen (2010b), each concept should be viewed not as an isolated unit, but as a representation of the conceptual structure inherent in the overall field under investigation creating a “generative concept system” (Picht and Draskau, 1985, p. 63).

In advancing the process of concept analysis, we adopted an interpretative approach at all moments of classification and description of concepts. In line with Walker and Avant (2005), the inductive process of concept synthesis led us to progressively identify relevant categories around which the concept of leadership in smart working contexts could be arranged. These categories were: (1) agile philosophy, (2) smart working settings, (3) advanced information technology, and (4) new employees’ expectations. These emergent subjects were jointly evaluated and discussed by the authors with the definitive aim of developing an overarching conceptualization of leading in smart working contexts.

## Scope Conditions: The Evolving Dynamics of the Smart Working Approach

**FIGURE 1:**
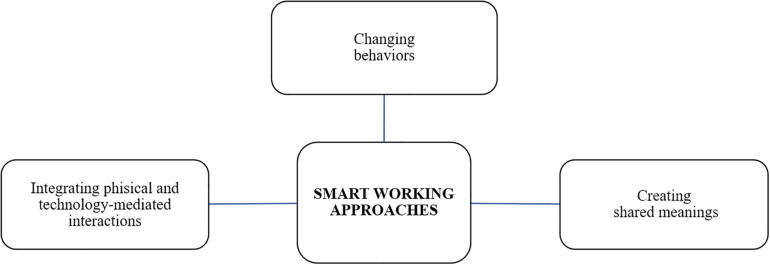
The evolutive dynamics of smart working approaches.

Smart working approaches combine flexibility, autonomy, and collaboration to achieve improved organizational performance and working environments (Zheltoukhova, 2014). These approaches strongly affect working and relational dynamics, especially with regard to the manager – employee relationship. As Figure 1 shows, the evolving dynamics generated by smart working can essentially be understood according to three main aspects: (1) changing behaviors, (2) creating shared meanings in change management processes, and (3) integrating physical and technology-mediated interactions. Below, we discuss each in turn in order to associate each aspect with the notions of leadership to which it is best suited.

The first aspect is the radical change that smart working requires in terms of observable, visible, verbal, and nonverbal behaviors that people adopt in the organizational setting. From the employee perspective, people must possess adequate computer or technology skills in order to maximize the potential of smart working. However, becoming a “smart worker” requires autonomy and empowerment; outcome-focused approaches; flexible time and space to work; trust; and collaboration. New ways of working also imply a need to rethink physical workspaces, main objectives, and allocation of resources (Tagliaro and Ciaramella, 2016). Flexibility with respect to working time and locations has already been shown to increase employee morale, and has been linked to the concepts of work – life balance, satisfaction, and performance (Hill et al., 2001; Gajendran and Harrison, 2007). That is, when organizational settings become dematerialized, new spaces for co-working and sharing emerge. From the leader perspective, this entails knowing how to: (1) foster collaborative and open relationships; (2) empower employees through delegation mechanisms, education and training; (3) facilitate knowledge sharing; (4) meet employee expectations; (5) work ethically, and (6) lead virtual teams, overcoming obsolete command and control styles. In other words, by acquiring autonomy and competence, and perceiving significance in their work, smart workers should develop self-determined behaviors and a strong intrinsic motivation to work. To that end, agile, ethical, organic, participative, and transformational notions of leadership can be elicited to accomplish these aspirations (Garg and Krishnan, 2003).

The second aspect concerns the importance of a broader change management process, whose results depend on how people interpret phenomena and “make sense” of change (Weick, 1995; Tagliaro and Ciaramella, 2016). In this process of sensemaking, leaders may have a dual role. Together with employees, top and middle management contribute, through their interpretative systems, to producing meanings with respect to change (Thomas et al., 1993). This may result in different subsets of (potentially divergent) shared meanings, where change can inconsistently emerge from discordant interpretations (Dawson and Buchanan, 2005). Dissonance may occur, resulting in needless messages, separation from the mission, and off-balance and poor performance (Goleman et al., 2013). In line with Weick (1988), many studies have shown that leaders who strive to create shared meanings of change and pay attention to employees’ emotions are more likely to succeed in the desired change (e.g., Gioia and Chittipeddi, 1991; Sanchez-Burks and Huy, 2009). By allowing for sensemaking and shared meanings, leaders work as architects of the context in order to avoid individual interpretation resulting in conflict with the overall vision of the organization (Bailey et al., 2017; Bednar and Welch, 2019). The promotion of a positive spiral enables the abovementioned triple-win configuration to occur, incorporating notions of purposeful leadership, resonant leadership, and emotional intelligence.

The third aspect concerns the impact of technology on worker–leader relationships. The use of digital technologies allows for remote working, supports more flexible work activities, and facilitates collaboration and knowledge sharing among employees (van Heck, 2010; Schallenmueller, 2016; Bednar and Welch, 2019). Nevertheless, smart working contexts are not centered on technology – although technology-based interactions constitute a common trait. While technologies enable more democratic and flatter workplaces, they may obscure certain ethical matters, such as forms of control over workers and a lack of transparency toward managers (Bednar and Welch, 2019). Furthermore, the dynamic nature of smart working makes it different from more “static” forms of remote e-working. Smart working includes both face-to-face and virtual interactions, in both physical and digital places of work. As result, the notions of e-leadership, smart leadership, and leadership 4.0 seem to fit one another well.

## Leading People in Smart Working Contexts

The collection of data from different sources led us to strictly relate smart working contexts to the agile philosophy; that is, uncertain environments in which agile leaders face fast-changing business contexts (McPherson, 2016). Thus, smart working implicitly requires leaders with mastery of the visionary and facilitative orientation, to the extent that they should inspire a vision and lead people to transform it into reality; characteristics that are found in what has been defined as the “catalyst level of agility” (Joiner and Josephs, 2007). A catalyst leadership culture includes high levels of participation, empowerment, and teamwork, where leaders work to change people’s behaviors “in ways that are beneficial to the organization and themselves” (Joiner, 2009, p. 32). This sense of guidance, which aims to establish a strong fit between people and organizational values, is also the central tenet of purposeful leadership, whose inspiring vision and commitment to stakeholders contribute to eliciting better results from employees (Yarlagadda et al., 2017). According to Goleman et al. (2013), leaders with an “intellectual” philosophy want to understand people, things, and the way they work by relying on cognitive competences and providing emotional security with respect to the future. Similarly, resonant leadership aligns with the notion of smart leadership. From the Latin *resonare*, this concept applies the synchronous vibration of sounds to people sharing wavelengths of emotions. In this sense, resonant leaders use empathy to reinforce emotional synchrony and, by connecting with others, make work more meaningful, provide support for overcoming uncertainties during change processes, and promote collective sensemaking. In other words, resonant leadership sustains the contagion of emotions and knowledge across their different organizational levels to sustain desired changes (Boyatzis, 2012). Beyond inspiring change, leaders in smart working contexts must imbue this change with sense. They act as role models, whose behaviors reflect values that organizations promote. According to the purposeful conception of leadership, it is important that employees are “ethically aligned – that is, see that their leader behaves ethically and also feel that their own values fit with that of their organization” (Yarlagadda et al., 2017, p. 29).

From the ethical perspective of leadership, leaders guide principled behaviors as they contribute to developing cognitive tools that lead employees to make the right choices. Leaders do this by using communication and reward systems and “aligning the multiple formal and informal systems” (Rok, 2009, p. 465). In that regard, people with a “humanistic” philosophy rely on personal relationships and human values, so that “they assess the worth of an activity in terms of how it affects their close relations” (Goleman et al., 2013, p. 122). Since the dynamic and flexible nature of smart working implies an “anywhere and anytime” logic of work, it is important to provide a cohesive environment in which people engage in achieving organizational goals. In this sense, having a “pragmatic” philosophy means believing that “usefulness determines the worth of an idea, effort, person or organization” (Goleman et al., 2013, p. 121). Although technology-mediated relationships can obscure the presence of a leader, this comfortable “absence” does not imply a laissez-faire leadership style – that is, a leader that completely avoids responsibilities and making decisions (Luthans, 2005). According to the organic approach, leaders should work as informal servants or facilitators of the group, where all team members share self-control and self-organizing principles in a general context of autonomy and trust (Rok, 2009). Therefore, trust must be built on principles of integration between different contributions to facilitate a collective result that is wider than that of the sum of individual contributions. These kinds of leaders are expected to appreciate the opinions of all group members (Finkelstein and Jackson, 2005); to be participative, by integrating the “hearts and minds” of all participants in the decision-making process; and to be transformational, by inspiring and activating employees beyond normal procedures (Bass, 1985; Rok, 2009). Transformational leaders aim to create and increase collaborators’ motivation and satisfaction with respect to their work. Focusing on shared values, they encourage people to contribute to achieving objectives (Burns, 1978) by overcoming the traditional concepts of time and workspace in favor of effectiveness in meeting targets. This overall flexibility is not a trivial interpretation: on the one hand, it is a stimulus to meet the expectations of smart workers; on the other, it can lead to difficulties in supervising, as well as feelings of isolation, workaholism, or overload when not handled properly. Moreover, the increased autonomy and job demands that smart working practices may place on employees require leaders to exhibit more coaching-oriented behaviors, by which individuals may be supported to find resources to manage their work tasks (Schwarzmüller et al., 2018). Taken together, these leadership approaches enable employees to develop strong intrinsic motivation and foster self-determined behaviors, thus pursuing psychological empowerment and improved work performance (Conger and Kanungo, 1988; Spreitzer, 2007).

With regard to the role of technology in enabling smart working environments, and considering the importance of combining both physical and digital places of work, there are two main points of interest here. One is that smart working contexts may require the presence of a broader leadership 4.0 culture. This is intended as “a fast, cross-hierarchical, team-oriented, and cooperative approach (to leadership), with a strong focus on innovation” (Oberer and ErKollar, 2018, p. 6), which allows for developing new mindsets, methods, and instruments; thus, when old responses to novel challenges no longer work, leaders must integrate culture and business processes to foster change (Pulley and Sessa, 2001). While the necessity of continuous innovation calls for speed and urgency, changes in attitudes and behaviors need time to mature. At first glance, this might seem like a paradox; however, it simply entails breaking down barriers that bind us to formal organization. Relationships must be built on authoritativeness rather than authority. Hung et al. (2010) referred to leaders as the architects of context, who play a central role in motivating and empowering employees to experiment and collaborate to generate new ideas. In this way, it is the network of relationships itself that changes. As this change occurs, the structure and so-called “centers of power” also change, in a process of continuous adaptation. According to Denison et al. (1995), in contexts characterized by complexity and uncertainty leaders must develop a behavioral repertoire that allows them to deal with inter-organizational, intra-organizational, and extra-organizational challenges (Sullivan et al., 2015). Drawing on an agent-based model, Sullivan et al. (2015) investigated leadership, networks, and innovation in order to specify generative mechanisms for the emergence of shared leadership structures over time and space. While digital technologies have provided a basis for new forms of organizing among geographically “complex multi-team systems” (Sullivan et al., 2015, p. 1), there is no doubt that greater connectivity is helping to break down traditional hierarchies (and organizational boundaries themselves), transforming workers’ activities within and outside of the workplace (Cortellazzo et al., 2019). The enabling power of technologies – especially those supporting communication, collaboration, and networks (as well as social networks, for which further investigation is needed), together with the increasingly pervasive diffusion of easy-to-use mobile devices – support individuals in sharing processes, even in real time and in different environments (Chudoba et al., 2005; Ahuja et al., 2007; Kim and Oh, 2015; Raguseo et al., 2016). The second point concerns how to elicit enthusiasm and inspire followers electronically; to build trust with employees who may never see their leader face to face; and to communicate effectively with dispersed team members (DasGupta, 2011). In that regard, the literature on e-leadership has offered important insights. The basic aim of e-leadership is to produce changes in the thinking, behaviors, and performance of individuals (Avolio et al., 2000). Avolio et al. (2014, p. 107) defined e-leadership “as a social influence process embedded in both proximal and distal contexts mediated by Advanced Information Technologies that can produce a change in attitudes, feelings, thinking, behavior, and performance.” In a similar vein, e-leadership is suggested as the most suitable form of leadership for flexible works, since it is uniquely able to combing both electronic and traditional methods of communication (De Leede and Heuver, 2016; Van Wart et al., 2019).

While addressing different notions of leadership with reference to smart working dynamics, the iterative process of analysis allowed us to identify and cluster the main antecedents, attributes, and outcomes of leadership in smart working contexts. The subsequent paragraphs provide a detailed description of these aspects.

### Antecedents

In our iterative process of literary analysis, we acknowledged four main conditions that allow smart leadership to occur. These conditions are: (1) agile philosophy, (2) smart working settings, (3) advanced information technology, and (4) employee’s evolving expectations. Interestingly, these facets operate at different levels (i.e., conceptual, operational, technological, and individual) to elicit the necessary presence of smart leadership behaviors in smart working contexts. Moreover, while the first two antecedents are more explanatory in terms of why leadership is important in smart working contexts, and what kind of role it plays, the last two antecedents advise on how leadership should occur in smart working contexts.

From a conceptual perspective, the agile philosophy and its principles are identified as the starting point. The *Agile Manifesto* (Beck et al., 2001) calls for new approaches that “emphasize people over process, software over documentation, customer collaboration over contract negotiation and responding to change over following a plan” (Medinilla, 2012, p. 38). In that regard, both the uncertainty of business environments and the numerous adaptive changes that “becoming agile” requires represent preliminary conditions that indicate the need for leaders of such change management processes. In operational terms, the agile philosophy supports the diffusion of smart working practices that aim to align and satisfy customers, employees, and organizations in the so-called triple-win configuration. However, this progressive convergence of interests and objectives is possible only if it is somehow “guided” and inspired. Again, this condition suggests the importance of leadership in smart working contexts. Moreover, the pervasive diffusion of advanced information technologies to support smart working practices has had a strong impact on the leader – follower relationship, thus preventing the full application of previously well-established leadership practices. Finally, employees’ expectations have also undergone a profound transformation, as workers have come to search not for instructions but for inspiration; they desire not simply extrinsic rewards but intrinsic motivation, professional growth, creativity, innovation, and better work – life balance. These conditions have led to radical change, which has made it necessary to enrich and further develop existing notions of leadership.

### Attributes

**FIGURE 2:**
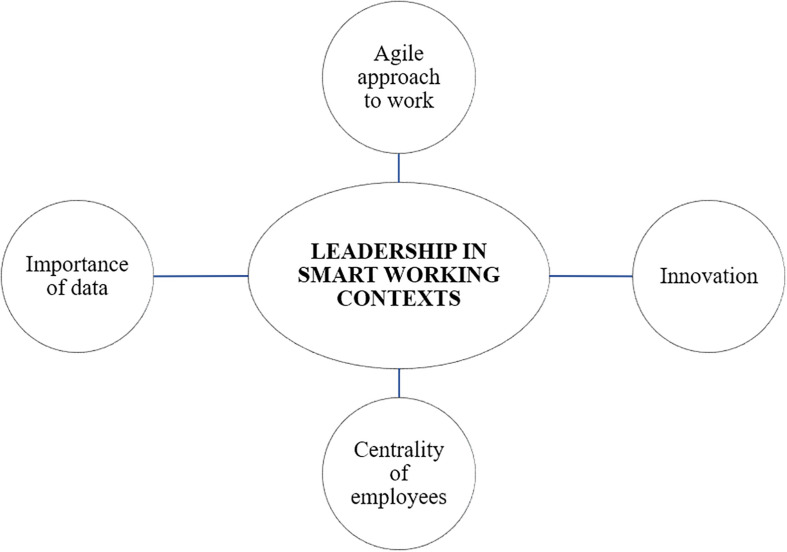
Leadership in smart working contexts.

With respect to attributes, eight essential elements of smart leaders emerged from the concept synthesis process. They are: (1) visioning; (2) inspiration; (3) self-awareness; (4) relationships creator; (5) lifelong learning; (6) execution; (7) innovation; and (8) ethical. They are showed in Figure 2.

The first is visioning, as having a vision of the future is important to understand the possible impacts of what is happening in the present and the ways in which to operate so as to successfully navigate this future. Second, smart leaders are expected to be inspiring, as they represent behavioral models that are to be followed by others. An inspiring vision is also fundamental when the aim is to create a shared meaning of change. The third attribute is self-awareness, which identifies the ability to be conscious of one’s own character and feelings. The fourth attribute is being a relationship creator, as smart leaders must combine technological and human aspects of relationships by creating a climate of trust and transparent communication. With the aim of continuously enhancing employees’ digital and soft skills, the fifth attribute is a predisposition for lifelong learning, as this motivates continuous improvement and supports an effective work across the team, while the sixth attribute, a high degree of execution, is important for ensuring the effectiveness of business actions. The seventh attribute is being an innovation ambassador, to the extent that smart leaders should not be limited to managing change but should create it by supporting strong innovation in thinking about work. When speaking about leadership agility, Joiner and Josephs (2007, p. 66) underline the importance for leaders to use verifiable data to solve problems and to “rethink issues and arrive at innovative solutions by taking what was successful in one context and applying it to another.” In that regard, Neubauer et al. (2017, p. 20) argue that agile leaders should “understand the value of using digital technologies to gather and analyze data,” by always looking “for new data sources to support informed decision-making.” While this data-driven approach supports leader’s adaptability and long-term vision (Neubauer et al., 2017), it determines two main effects. On the one hand, as noted by Cortellazzo et al. (2019), it will lead leaders to increasingly collaborate with IT manager in order to orientate data analysis and make sense of results (Harris and Mehrotra, 2014; Vidgen et al., 2017). On the other hand, it will require to create more ethically driven contexts – representing the eighth attribute – in which smart leaders use data for improving processes or analyzing areas of interventions, and avoid utilizing technological devices to control or study workers’ behaviors.

### Outcomes

Starting from the antecedents and attributes of leadership in the smart working context, our conceptual analysis definitively identifies four main consequences that should follow smart leadership. These outcomes are: (1) new mindset, methods, and instruments; (2) psychological empowerment; (3) improved work performance; and (4) person – organizational alignment.

First, when leadership is correctly enacted and supported in smart working contexts, it contributes to developing a new mindset in terms of thinking, attitudes, and behaviors. In doing so, it allows employees to be more supported in the adoption of new methods and new instruments of work. Second, if attributes of leadership are well implemented, they encourage employees’ intrinsic motivation, critical awareness, self-control, and, more generally, self-determined behaviors. Taken together, these first two outcomes result from the intrinsically facilitative function of leadership in smart working contexts. In this sense, they can be understood as intermediate-level outcomes.

Looking at the performative function of leadership in smart working contexts, we identified two further advanced-level outcomes. On the one hand, smart leaders may leverage the intermediate-level outcomes to foster enhanced person – job fit. In this way, as predicted by several studies in the reference literature, the better the alignment between job demand and employees’ attitudes and desires, the better the work-related outcomes (e.g., Caldwell and O’Reilly, 1990; Edwards, 1991). On the other hand, leaders must connect work performance and organizational objectives. If this connection is actually achieved, the definitive outcome of leadership in smart working contexts will be the expected alignment between individuals and the organization.

## Concept Synthesis

The interpretative moment of concept synthesis entailed summarizing all identified themes into a minimum number of categories, upon which an overarching conceptualization of leading in smart working contexts can be developed. As a result, we found four key concepts that describe the essence of this construct. These concepts are (1) agile approach to work, (2) innovation, (3) centrality of employees, and (4) importance of data.

Therefore, we define “smart leadership behavior” as facilitative behavior that naturally emerges in smart working contexts to inspire change and produce innovation in ways of thinking about work. While using advanced technologies to improve organizational effectiveness, such leadership should put people at the center of all processes, which should be consistently organized around the principles of agility, autonomy, trust, and responsibility. The inspiring vision outlined by leaders, together with ethically driven behaviors, contributes to creating a shared meaning of change, thus resulting in the alignment of both individual and organizational values, objectives, and interests. In this way, smart leadership enables a triple-win configuration in which employee, customer, and organizational needs are satisfied. Table 1 synthesizes the entire process and the main findings of concept synthesis.

**TABLE 1:** A comprehensive view of leadership in smart working contexts.

Concept synthesis method	Field delimitation	Smart working dynamics		
		
		Changing behaviors	Creating shared meanings	Integrating physical and technological interactions
	**Sources collection**	Agile leadership Ethical leadership Organic leadership Transformational leadership	Purposeful leadership Resonant leadership Emotional intelligence	Leadership 4.0 e-leadership Smart leadership

	**Notions of leadership**			

**Categories and subcategories**	**Antecedents**	**Attributes**	**Outcomes**	

	Agile philosophy Smart working settings Advanced Information Technology New employees expectations	Visioning Inspiration Self-awareness Relationships creator Lifelong learning Execution Innovation Ethical	New mindset, new methods, new instruments Psychological empowerment Better work-related outcomes Person-Organization alignment	Facilitative function Performative function

**Evaluation**	**Leadership in smart working contexts**

	A facilitative behavior that naturally emerges in smart working contexts to inspire change and produce innovation in the ways of thinking about work. While using advanced technologies to improve organizational effectiveness, it should put people at the center of all processes, which are consistently organized around the principles of agility, autonomy, trust and responsibility. Its inspiring vision, together with ethically driven behaviors contributes to create a shared meaning of change, thus resulting into the alignment of both individual and organizational values, objectives and interests

	Agile approach to work	Innovation	Centrality of employees	Importance of data

*Source: Own elaboration.*

## Discussion

According to our conceptualization, the notion of leadership in smart working contexts does not represent a new idea; rather, we want to scientifically depict such leadership as a naturally emerging behavior that combines agile logics and change management practices to align interests at different levels of the organization. Instead of making the list of leadership taxonomies even longer, the aim of this research is essentially to propose a notion of leadership that can be explicitly applied to smart working contexts. In line with our premises, we conclude that the concept of smart leading is an intrinsically composite construct that benefits from both older and more recent notions of leadership to respond to the changeable needs of smart working practices. To that end, our arguments suggest that the facilitative and performative functions of leadership are essential to pursue a dual alignment – at the person – job level and at the person – organization level – that might lead to the alleged triple-win configuration of smart working approaches.

However, a critical aspect concerns the fact that, while the natural emergence of this kind of leadership is well suited to the agile approach, it implicitly assumes that smart working contexts should foster the development of informal structures of leadership (and, more importantly, that actors in these contexts are willing to do so). From a managerial perspective, this means that if hierarchy-based leadership structures do not converge with those that arise informally when smart working is activated, the alleged change may fail due to conflicts, resistance, and disorientation among the people involved. Starting from the contribution of Trentini (2003), we find that formal leaders are more closely associated with the notion of “manifested” and “alleged” roles (Jaques, 1994), since formal leadership is strictly related to the bureaucracy of organizational charts and job positions, while informal leaders tend to be aligned with “real” and “adequate” roles (Jaques, 1994), as they represent a kind of guide for people, and enable teams to work to the best of their ability. In that sense, the role of informal leaders is substantial and authentic. Moreover, the existence of informal structures has a strong impact on the consolidation of organizational networks that smart working contributes to shaping. Thus, smart working facilitates the building of networks in which people feel freer from hierarchical constraints, communicate better, and work collaboratively and with greater autonomy. In such contexts, leaders are viewed not as imposed by organizational charts, but as part of the network itself. From this perspective, smart leaders could actually act as facilitators of the network and therefore foster organizational change (Trentini, 2003). Finally, when organizations develop a culture based on trust and continuous learning, they contribute to creating an “exchange” of leadership, which will lead to greater autonomy and self-management capacity of individuals and groups (Bierly et al., 2000; Wang and Ahmend, 2002). Therefore, headship structures should be progressively substituted with leadership structures that emerge informally. In this way, leaders can opportunely manage job relations that are based on knowledge sharing and wide participation, rather than on the mere responsibility chain.

Taken together, these arguments suggest that smart working environments should be accompanied by a deepened recognition of emerging informal leadership structures – for a number of reasons. First, informal structures evolve over time and are flexible, and thus have greater capacity to pursue the agility of smart working contexts. Moreover, while formal structures are based on hierarchical positions, informal structures emerge from the subjectivity of human interactions and job relations (Trentini, 2003; Busetti, 2018). Being the result of human relations, they naturally foster participation, autonomy, and knowledge sharing. What results is the creation of a new type of network in which communication and interaction are facilitated by smart leaders. For this reason, although smart working is enabled by technologies, smart leadership is designed to be strongly centered on the empowerment of people. This statement summarizes, in a single answer, the evidence resulting from our three research questions, highlighting the triple-win configuration that sees customers, employees, and organizations involved in a positive spiral.

## Concluding Remarks

Starting from a lack of studies that have specifically addressed the role and the characteristics of leadership for smart working purposes, this paper applied a concept synthesis methodology to progressively propose the notion of leading in smart working contexts. Without dwelling on providing a new definition of smart leadership – a concept that is constantly changing and evolving – it aimed at a concept delineation that contributes to make the idea more understandable and practically applicable to smart working contexts.

Overall, this paper offers several insights for both research and managerial audiences. Through an iterative process of literary analysis, our study critically analyzes and compares different notions of leadership to evaluate the adequacy of the scope conditions of smart working practices. Moreover, by delineating antecedents, attributes, and outcomes that characterize leadership in smart working contexts it contributes to proposing a more operationalizable description of the concept. Our methodological approach allowed us to identify the nature and extent of research evidence concerning the topic of leadership in smart working contexts. Our study’s qualitative derivation meant that there could not be predetermined rules for proceeding with the analysis. However, this does not represent a limitation, since the centrality of interpretation is inherent in the very nature of the approach. The further we proceeded, in depth and in detail, the greater the consistency that evolved between the inclusion and exclusion criteria and the background of the team of researchers involved. These characteristics were relevant not only to the analyses carried out individually, but also to the subsequent moments of classification, description of concepts, and interpretative synthesis, as was the number of people involved. Our utilization of a flexible method should be considered a strength and a source of cognitive wealth in this research. Flexibility is, in fact, one of the major strengths, from a methodological standpoint, of explorative studies, which makes them conceptually applicable to many purposes by combining elements of terminological analysis and elements from theoretical, academic, empirical, and/or popular works.

For scholars, this work represents a first attempt to refine, categorize, and systematize a wide range of notions related to the concept of smart leadership. Our findings may represent a starting point for conducting future research aimed, for instance, at developing more extended conceptual frameworks, conducting systematic reviews of the literature, or performing empirical studies to address the effective application of the concept in terms of “typical” or “deviant” models that can be detected from managerial action. To that end, future empirical investigations could include qualitative inquiries such as single case studies, comparative case studies, ethnographies, or other quantitative or mixed research strategies (such as bibliometrics, qualitative comparative analysis, structural equation modeling). From a theoretical perspective, it would be interesting to deepen the relationship between antecedents, attributes, and outcomes of leadership in smart working contexts, as well as to understand, predict, and monitor it at different levels. While the individual level of analysis should focus on the relationship between the individual leader and her/his followers, at a group level researchers might investigate the relationship between the leader and the collective group of followers, communities of practice, and power groups; at the organizational level, it might be pertinent to focus on structures; organizational processes; and practices, values, and culture.

From a managerial perspective, our findings provide a comprehensive understanding of what “being a smart leader” actually means, and how to operatively apply this conceptual notion in smart working contexts. In terms of action, this means understanding how to shape attributes and capabilities of leaders; the values and principles around which teams should be organized; how to manage the relationship between formal and informal structures of leadership; and how to enable the convergence of employee, customer, and organizational interests in a triple-win configuration – which is the definitive aim of smart working approaches. Moreover, our arguments suggest that the facilitative and performative functions of leadership are both important in pursuing the expected outcomes. Since the presence of facilitating behaviors enables effective change, individual knowledge, skills, and competences must be supported in parallel via enabling technological infrastructures, which remain necessary but not sufficient conditions to complete the definition of smart leadership. The combined disposition of complementary variables should be recognized, in line with the contingent conditions of operation (Corso et al., 2014). From this perspective, our work suggests that effective smart leadership, based on the four main defining attributes (innovation, people, data, and agile logics), becomes essential when the aim is to implement smart working approaches in a successful direction.

## Author Contributions

MI and GM contributed to the conception of the study. MI contributed to designing the study, wrote the sections “Introduction,” “Scope Conditions,” “Antecedents,” “Outcomes,” and “Concept Synthesis,” and created the table. MI and CM wrote the sections “Research Rationale and Methods” and “Concluding Remarks.” All authors contributed to collecting sources, organizing materials, analyzing literary documents and writing the remainder of the manuscript, contributed to manuscript revision, and reading and approving the submitted version.

## Conflict of Interest

CM was employed by the company IterEgo at the time of publishing this work. The remaining authors declare that the research was conducted in the absence of any commercial or financial relationships that could be construed as potential conflicts of interest.
